# Pirfenidone gel in patients with localized scleroderma: a phase II study

**DOI:** 10.1186/s13075-014-0510-4

**Published:** 2015-01-28

**Authors:** Marco Rodríguez-Castellanos, Alberto Tlacuilo-Parra, Sergio Sánchez-Enríquez, Ezequiel Vélez-Gómez, Elizabeth Guevara-Gutiérrez

**Affiliations:** Dermatology Department, Instituto Dermatológico de Jalisco, Secretaria de Salud Jalisco, Avenida Federalismo 3102, ZP 45190 Zapopan, México; UMAE Hospital de Pediatria CMNO, IMSS, Monte Olimpo Número 1413, ZP 44340 Guadalajara, México; Biochemistry Laboratory, CUCS, Universidad de Guadalajara, Siera Movada 950, ZP 44340 Guadalajara, México; Department of Pathological Anatomy, Nuevo Hospital Civil de Guadalajara, OPD, Salvador Guevero y Zubieta 750, ZP 44340 Guadalajara, México

## Abstract

**Introduction:**

Localized scleroderma is an inflammatory disease in its first stages and a fibrotic process in later stages, principally mediated by the transforming growth factor β. To date, there is no standard treatment. The objective of this study was to determine the effectiveness and safety of 8% pirfenidone gel in patients with localized scleroderma.

**Methods:**

This was an open phase II clinical trial that included 12 patients. Treatment with pirfenidone was indicated, three times daily for 6 months. Patients were evaluated clinically with the modified Localized Scleroderma Skin Severity Index (mLoSSI), as well with a durometer and histologically using hematoxylin and eosin stain and Masson’s trichrome stain.

**Results:**

The baseline mLoSSI average scores were 5.83 ± 4.80 vs. 0.83 ± 1.75 (*P* = 0.002) at 6 months. The initial durometer induration of the scleroderma plaques was 35.79 ± 9.10 vs. 32.47 ± 8.97 at 6 months (*P* = 0.05). We observed histopathological improvement with respect to epidermal atrophy, inflammation, dermal or adipose tissue fibrosis and annex atrophy from 12.25 ± 3.25 to 9.75 ± 4.35 (*P* = 0.032). The 8% pirfenidone gel application was well tolerated, and no side effects were detected.

**Conclusions:**

This is the first study on the therapeutic use of pirfenidone gel in localized scleroderma. It acts on both the inflammatory and the fibrotic phases. Considering its effectiveness, good safety profile and the advantage of topical application, pirfenidone is a treatment option in this condition.

## Introduction

Localized scleroderma is a disease with no known cause and varied clinical expression that is characterized by sclerosis of the skin [1]. Its pathogenesis is centered on autoimmune alterations with a distinctive cytokine profile. It is an inflammatory disorder during its first stages, evolving to a fibrotic process principally mediated by tumor necrosis factor α (TNF-α), transforming growth factor β (TGF-β), connective tissue growth factor (CTGF) and platelet-derived growth factor (PDGF) [2]. There is no standard treatment for this disease, but the actual tendency suggests that treatments based on pathogenesis are preferable [2].

Pirfenidone is a molecule derived from pyridone (5-methyl-1-phenyl-2-[1*H*]-pyridone) and has been used as an antifibrotic agent [3]. Its capacity to suppress the expression of the *TGF-β* gene at the transcriptional level has been demonstrated, along with a dose-dependent reduction of collagen types I and III mRNA levels [4,5]. Also, it can affect the cascade of the inflammatory and fibrotic processes, suppressing TNF-α induction, a fundamental molecule in inflammatory and immunological processes [6]. In general, the safety profile of pirfenidone is favorable, and significant toxicity has not been reported [7,8].

Taking into account these anti-inflammatory and antifibrotic properties, our objective was to evaluate the safety and effectiveness of 8% pirfenidone gel in patients with localized scleroderma.

## Methods

This study was an open phase II clinical trial approved by the local ethics and research committee of the Instituto Dermatologico de Jalisco. Patients included both sexes, were 18 years old or older and had a clinical and histopathological diagnosis of active localized scleroderma and no topical or systemic treatment for 2 or 4 weeks prior to the study, respectively. Patients gave their informed consent to participate, a case history was taken and the following assessments were done: (1) modified Localized Scleroderma Skin Severity Index (mLoSSI) [9], (2) cutaneous induration, (3) visual analogue scale, (4) punch biopsy and (5) safety evaluation.

### Assessments

#### Modified Localized Scleroderma Skin Severity Index

The mLoSSI is a 0- to 162-point scale (the higher the score, the greater the severity). It includes an evaluation of erythema, cutaneous density and presence of new lesions or extension of the previous ones, using a scale of 1 to 3. A basal evaluation was done and then repeated at 3 and 6 months of treatment.

#### Cutaneous induration

Cutaneous induration was measured with a durometer (Rex Gauge Type 00; Rex Gauge, Buffalo Grove, IL, USA) [10,11] using a 0 to 100 durometer unit (DU) scale, with a higher score indicating greater induration. Three evaluations were done on each lesion, at three distinct points, and the average of the measurements was used for the analysis. The measurements were also done on an unaffected contralateral area, with a basal evaluation and repeated at 3 and 6 months of treatment as a quality control measure.

#### Visual analogue scale

For the patient’s evaluation of the effectiveness of treatment, we used a visual analog scale (VAS), which gives a score of 0 to 10 with respect to skin hardness and presence of itchiness, where 0 is the absence of symptoms and 10 is the maximum perception of these symptoms. This was done at the beginning and end of treatment.

#### Punch biopsy

Punch biopsies were performed at the start and end of treatment. The final biopsy was done next to the initial biopsy position. The obtained tissue was stained with hematoxylin and eosin stain and Masson’s trichrome stain, with a blind examination by an expert pathologist. The evaluated parameters included epidermal atrophy; papillary dermis, reticular dermis or adipose tissue fibrosis; and atrophy of the annexes. A semiquantitative, four-grade scale was designed: 0 = normal, 1 = slight, 2 = moderate and 3 = severe. Table 1 lists microscopic characteristics that were used as parameters to evaluate the effectiveness of pirfenidone on localized scleroderma lesions.

**Table 1:** **Histological assessment criteria** ^**a**^

**Epidermal atrophy**			**Papillary dermis fibrosis**		
Normal	0	More than seven layers	Normal	0	Absence
Light	1	Six to seven layers	Light	1	Reduction in thickness of lax tissue
Moderate	2	Four to five layers	Moderate	2	Segmental hyalinization
Severe	3	Three layers or less	Severe	3	Complete hyalinization
**Dermal and subcutaneous infiltration**			**Reticular dermis fibrosis**		
Normal	0	Absence	Normal	0	Absence
Light	1	Isolated lymphocytes	Light	1	Small pockets of hyalinization
Moderate	2	Small groups of lymphocytes	Moderate	2	Presence of moderate hyalinization
Severe	3	Large groups of lymphocytes	Severe	3	Total hyalinization
**Adipose tissue fibrosis**			**Annex atrophy**		
Normal	0	Absence	Normal	0	Absence
Light	1	Light thickening of adipose septa	Light	1	Periadnexal fibrosis
Moderate	2	Moderate thickening of adipose septa	Moderate	2	Reduction in size of annexes
Severe	3	Severe thickening of adipose septa	Severe	3	Presence of adnexal vestiges

^a^Microscopic characteristics that were used as parameters to evaluate the effectiveness of pirfenidone on localized scleroderma lesions.

#### Safety evaluation

For the safety evaluation, laboratory tests were done (complete blood count, liver function tests and blood chemistry) to obtain basal levels, and then these tests were repeated at the first, third and sixth months of treatment and compared with baseline values. Local side effects, adverse clinical events and abnormal laboratory results were evaluated at the beginning and at 1, 3 and 6 months of treatment.

### Treatment

Patients were instructed to apply 8% pirfenidone gel three times daily for 6 months using the standard fingertip unit (0.5 g for an area of 100 to 120 cm^2^) [12].

### Statistical analysis

Statistical analysis was done with SPSS version 19.0 software (IBM, Armonk, NY, USA). The Wilcoxon rank-sum test was used to compare the pre- and posttreatment evaluations. A *P*-value *<*0.05 was considered statistically significant.

## Results

A total of 12 patients with localized scleroderma were included, 9 (75%) of whom were women and 3 (25%) of whom were men. Their mean age was 46 years old. Circumscribed, localized scleroderma was present in three (25%) patients, linear in three (25%) patients and the generalized variant in six (50%) patients. The sizes of the lesions ranged from 0.3 cm^2^ to 765 cm^2^ (Table 2).

**Table 2:** **Epidemiological data** ^**a**^

**Patient**	**Sex**	**Age (yr)**	**Time of evolution (yr)**	**Previous treatment**	**Type of scleroderma**	**Size of smallest lesion (cm** ^**2**^ **)**	**Size of largest lesion (cm** ^**2**^ **)**	**Affected segments**
1	F	47	10	Yes	Linear	10.5	97.5	1
2	F	24	1.5	No	Linear	1.5	17.5	1
3	F	77	3	Yes	Generalized	1	412.5	6
4	F	66	6	Yes	Generalized	2	255	5
5	F	54	3	Yes	Generalized	31.5	448	3
6	F	29	3	Yes	Circumscribed	360	765	2
7	F	21	10	Yes	Circumscribed	45	52.2	1
8	M	31	8	No	Circumscribed	0.48	7	1
9	M	39	3	Yes	Generalized	0.25	136	13
10	F	46	2	Yes	Circumscribed	12	22.5	1
11	M	51	<1	No	Generalized	1	36	3
12	F	48	4	No	Generalized	6	189	2
Mean	NA	46.5	3	NA	NA	4	116.75	2
Maximum	NA	77	10	NA	NA	360	765	13
Minimum	NA	21	1.5	NA	NA	0.25	7	1

^a^The patients were predominately female (75%) and had received previous treatment (67%), and one-half (50%) had the generalized type of the disease. NA, Not applicable.

**Figure 1:**
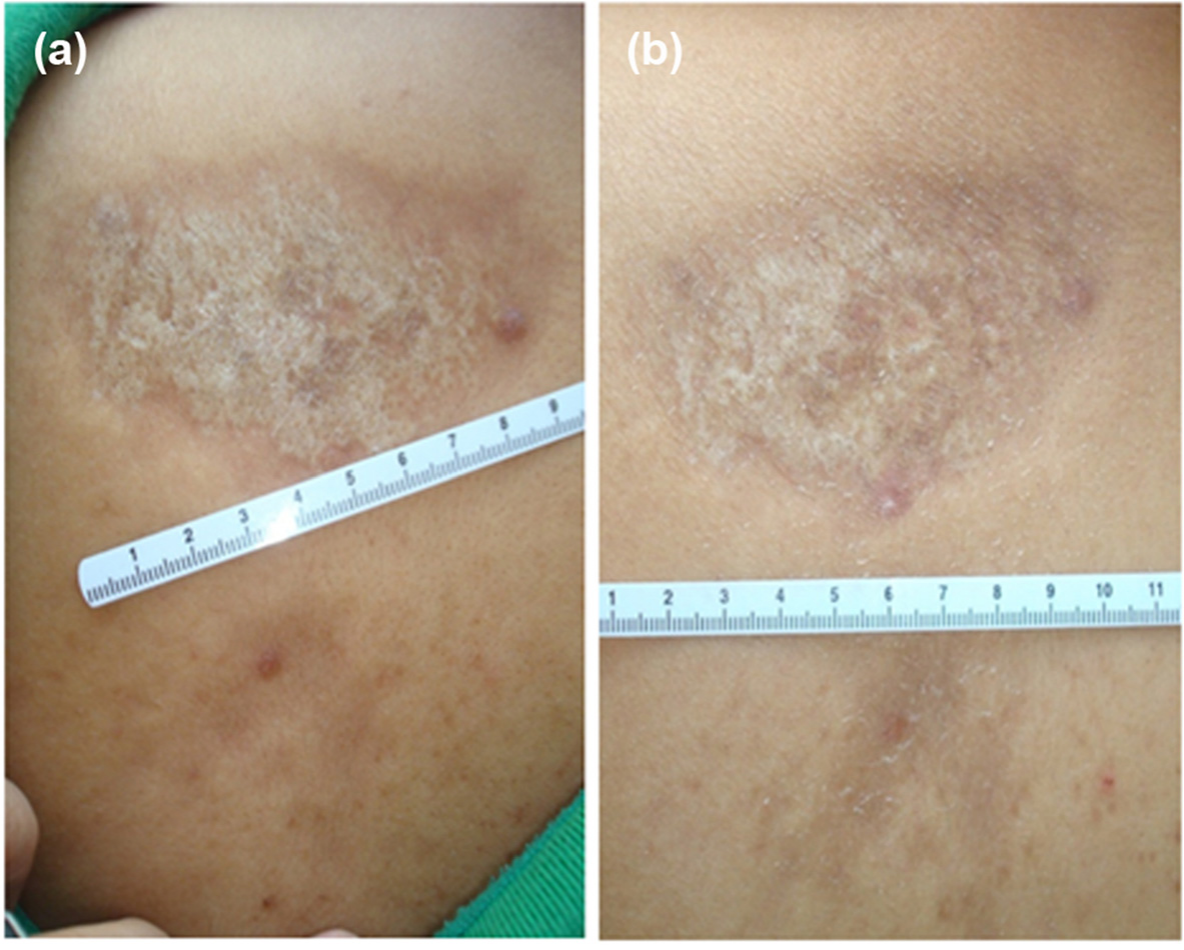
**Clinical changes before and after treatment. (a)** Note the ivory-white color at the beginning of the treatment. **(b)** Reduction in color and size at the end of treatment.

**Figure 2:**
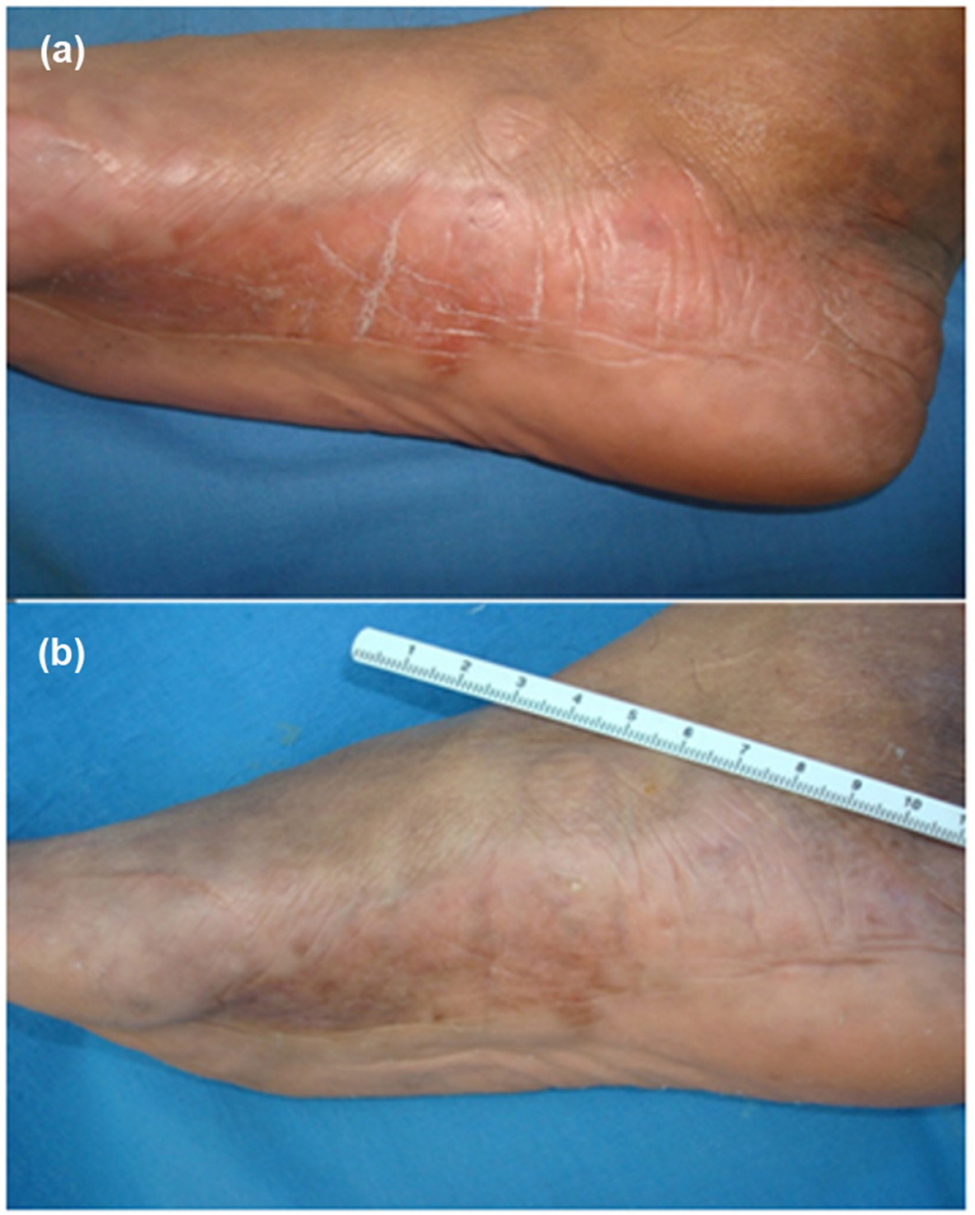
**Scleroderma erythema, peeling and fissures remission. (a)** Scleroderma plaque with erythema, peeling and fissures. **(b)** Remission of the signs with a better state of hydration; only residual hyperpigmentation can be observed.

All patients showed a sustained improvement in their lesions throughout the study. The basal mLoSSI average was 5.83 ± 4.80 vs. 1.33 ± 1.92 at 3 months of treatment (*P* = 0.003) vs. 0.83 ± 1.75 at 6 months (*P* = 0.002) (Figures 1 and 2, Table 3). The induration of the scleroderma lesion compared with the induration of the contralateral area, which served as a control, had a mean basal measurement of 35.79 ± 9.10 DU (24.18 to 56.73 DU) vs. 26.86 ± 8.51 DU (13.06 to 41.36 DU) for normal skin. At 3 months of treatment, the mean was 33.80 ± 7.24 DU (18.74 to 42.81 DU) vs. 26.90 ± 7.24 DU (12.38 to 35.03 DU) for normal skin (*P* = 0.919), and the mean at the end of treatment was 32.47 ± 8.97 DU (13.51 to 49.12 DU) vs. 27.06 ± 8.50 DU (10.85 to 38.56 DU) of normal skin (*P* = 0.386).

**Table 3:** **Effectiveness clinical variables** ^**a**^

**Patient**	**Initial mLoSSI**	**Final mLoSSI**	**Improvement mLoSSI (%)**	**Mean basal durometer**	**Mean final durometer**	**Improvement durometer (%)**	**Initial VAS hardness**	**Final VAS hardness**	**Improvement VAS hardness (%)**	**Initial VAS pruritus**	**Final VAS pruritus**	**Improvement VAS pruritus (%)**
1	1	0	100	56.73	49.12	13.41	0	4	−4	0	5	−5
2	3	0	100	35.6	34.51	3.06	0	0	0	0	0	0
3	14	6	57	36.95	38.09	−3.08	7	5	29	0	0	0
4	11	1	93	28.37	30.27	−6.7	8	2	75	7	2.5	65
5	9	0	100	33.71	31.14	7.62	5	3	40	8	3	62
6	3	0	100	26.21	27.03	−3.13	6.5	1.5	77	6.5	2	69
7	2	0	100	32.49	35.39	−8.93	10	4.5	55	5	2.5	50
8	2	1	50	30.23	26.26	13.13	5	3	40	0	0	0
9	4	2	50	45.52	42.02	7.69	7	3	57	5	2	60
10	3	0	100	42.68	35.29	17.31	8	0	100	5	0	100
11	14	0	100	36.83	27.05	26.56	0	0	0	3	0	100
12	4	0	100	24.18	13.51	44.12	3	3	0	8	0	100
Mean	5.83	0.83	NA	35.79	32.47	NA	4.96	2.42	NA	3.96	1.42	NA
*P*-value^b^		0.002			0.05			0.032			0.065	

^a^The response to pirfenidone is evidenced by improvement in both physician (mLoSSI and durometer units) and patient (hardness of the lesions) evaluations. ^b^Wilcoxon rank-sum test. mLoSSI, Modified Localized Scleroderma Skin Severity Index; VAS, Visual analogue scale.

**Figure 3:**
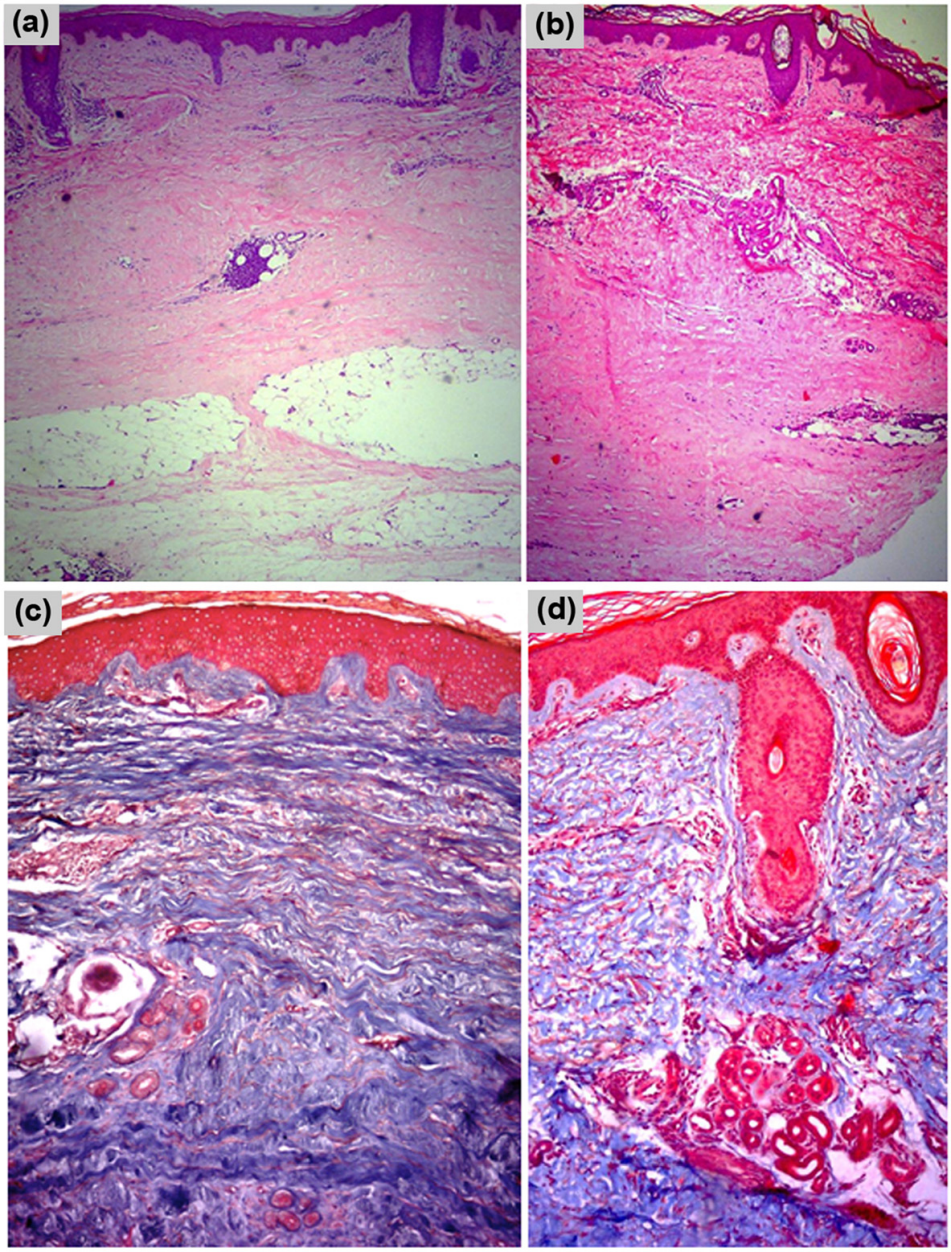
**Histologic changes before and after treatment. (a)** Pretreatment fibrosis, well-defined pockets of perivascular inflammation in the dermis and subcutaneous cellular tissue. **(b)** Posttreatment biopsy, collagen loosening of compaction in the superficial and middle reticular dermis with recovery of the annexes. **(c)** Masson’s trichromic stain before treatment. **(d)** Posttreatment biopsy showing the changes described previously (original magnification, 40×).

Patients’ assessments of hardness of the lesions and presence of pruritus showed improvement with the application of pirfenidone, although the difference was not significant (Table 3). At the beginning of the study, nine (75%) of the twelve patients stated that they had skin hardness, and, of these nine, one (8.3%) (patient 1 with linear scleroderma) felt that the hardness had worsened and another (8.3%) did not detect any improvement (patient 12 with generalized scleroderma). Eight (66.6%) patients reported pruritus, and one (8.3%) indicated worsening at the end of the study (patient 1). The mean basal global score for the histopathological alterations was 12.25 ± 3.25 (5 to 17) vs. 9.75 ± 4.35 (3 to 14) at 6 months of treatment (*P* = 0.032) (Figure 3, Table 4).

**Table 4:** **Histopathological evaluation criteria** ^**a**^

	**Epidermal atrophy**		**Dermal infiltration**		**Adipose tissue infiltration**		**Papillary dermis fibrosis**		**Reticular dermis fibrosis**		**Adipose tissue fibrosis**		**Adnexal atrophy**	
	**Basal**	**Final**	**Basal**	**Final**	**Basal**	**Final**	**Basal**	**Final**	**Basal**	**Final**	**Basal** ^**b**^	**Final** ^**c**^	**Basal**	**Final**
Absence	1	4	0	0	4	3	1	3	1	4	2	0	1	1
Light	1	4	4	8	3	9	6	3	4	2	3	3	4	3
Moderate	4	1	5	3	5	0	2	3	1	6	2	1	3	3
Severe	6	3	3	1	0	0	3	3	6	0	4	5	4	5
Mean (SD)	2.25 (0.96)	1.25 (1.21)	1.92 (0.79)	1.42 (0.67)	1.08 (0.90)	0.75 (0.45)	1.58 (1.0)	1.5 (1.17)	2 (1.13)	1.17 (0.94)	1.72 (1.19)	2.22 (0.97)	1.83 (1.03)	2 (1.04)
*P*-value^d^		0.010		0.034		0.305		0.705		0.039		0.276		0.581

^a^The microscopic findings showed statistically significant changes in epidermal atrophy, dermal infiltration and reticular dermis fibrosis. ^b^Nonevaluable case. ^c^Three nonevaluable cases. ^d^Wilcoxon rank-sum test. SD, Standard deviation.

The reported local side effects after applying pirfenidone were present in 11 patients (92%). These included slight, short-term burning sensation, which subsided after using a common lubricant, with no need to suspend or reduce the medication dosage. Additionally, there were no statistically significant changes in the laboratory tests before and after treatment.

## Discussion

It is difficult to assess the effectiveness of pharmacological agents in localized scleroderma, owing to various factors: rareness of the disease, heterogeneity of its severity in different population studies, difficulty in establishing controls, diverse modes of evaluation for improvement and absence of serological markers for activity [2,13,14].

Taking this into account, we decided to assess the therapeutic effectiveness of 8% pirfenidone gel using several evaluation tools that allowed us to demonstrate its usefulness in localized scleroderma by lowering of the mLoSSI score, reduction in the skin induration curve using a durometer, as well as with improvement in both the histopathological changes and (VAS) score, instruments that had not been used together as a set in previous studies.

The improvement in the localized scleroderma lesions with the application of 8% pirfenidone gel was evident with the mLoSSI score, showing an improvement in 87.5% of the treated cases and 100% improvement in 66% at 6 months of treatment (*P* = 0.002). This result is comparable to that observed with the application of 5% imiquimod cream, an interferon γ inductor and TGF-β inhibitor, with reduction of the skin induration and thickening in patients with localized scleroderma [15]. However, in the study of Dytoc *et al*., a subjective clinical scale was used to determine the changes in the disease and was not validated to assess the changes in pigmentation, induration, erythema and telangiectasias, which makes it difficult to reproduce their results. In contrast, we used the mLoSSI, a validated method to assess localized scleroderma with interobserver and intraobserver agreement of 0.70 and 0.77, respectively, and sensitivity to change in a 10-week period, making it a recommended tool for clinical studies as a validated clinimetric instrument [9].

We additionally demonstrated the usefulness of 8% pirfenidone gel with the durometer, a handheld device that quantitatively measures skin hardness. The measurements done with this device are simple, accurate, valid, objective, precise and sensitive to change [11,16]. Also, in this study, we did measures on control zones, which support the consistency of the observer’s assessment. The mean induration of the localized scleroderma plaques diminished between the basal and final measurements (35.79 DU vs. 32.47 DU, *P* = 0.05), showing a tendency toward reduction. We speculate that an increase in the treatment period with pirfenidone could obtain a statistically significant reduction.

The Kroft *et al*. study also showed the effectiveness of 0.1% tacrolimus for the treatment of active localized scleroderma inflammatory lesions. A significant difference was found between topical tacrolimus vs. petrolatum with respect to the scores for hardness, obtained using a durometer (*P* < 0.005) and clinical characteristics (*P* = 0.019) [10].

Few authors have included histopathological changes in their evaluations [15,17]. Our study showed histologic changes that were in accord with the clinical improvement, showing a statistically significant change in dermal infiltration (*P* = 0.034) and fibrosis of reticular dermis (*P* = 0.039), which could be explained by the anti-inflammatory and antifibrotic effects of pirfenidone [3,18,19].

The beneficial effects observed for 8% pirfenidone gel on localized scleroderma plaques may be related to its reported capacity to inhibit proinflammatory cytokines levels, as well as to activate interleukin 10 (IL-10) production. Clinical effects on inflammation and fibrosis have been recognized by Carter *et al*., on idiopathic pulmonary fibrosis, as well as by Armendariz-Borunda *et al*., in skin scarring caused by burns in children [3,18,20], but this cytokine change was not evaluated in the present work.

The mechanism of action of pirfenidone on localized scleroderma differs from that observed with other medications, such as topical tacrolimus, mycophenolate mofetil and betamethasone dipropionate/calcipotriol, which have shown effectiveness only in the incipient inflammatory stages of localized scleroderma [14,21-23]. Additionally, pirfenidone has an effect on the development of fibrosis [7,18,20].

The tolerability observed in the patients in this series is similar to that reported for topical use in pediatric patients with scarring secondary to burns [20]. Topical side effects were minimal and did not require suspending the medication, and no clinical or laboratorial adverse effects were observed.

Pirfenidone seems to have specific attributes for the treatment of localized scleroderma. It has shown antifibrotic effects in *in vivo* and *in vitro* models, reducing the production of cytokines such as TGF-β, PDGF, fibroblast growth factor β, the deposit and synthesis of collagen, inhibition of the recruitment and/or expression of extracellular matrix production cells has anti-inflammatory effects by inhibition of the liberation of proinflammatory cytokines TNF-α, IL-1β, IL-6, IL-8 and IL-12 and increased anti-inflammatory cytokines such as IL-10. Also, evidence exists that pirfenidone suppresses the production or liberation of chemotactic cytokines from inflammatory cells, reducing the accumulation in response to stimuli, and shows antioxidant effects with improved levels of enzymatic systems, such as the superoxide dismutase or the myeloperoxidase, along with its capturing effects of free radicals [3-5,8,18,19]. All these processes could be the mechanisms that mediated the results obtained in this study.

The limitations of our study include the small number of patients, the lack of a control group and the short duration of treatment. In future studies, the follow-up phase must be prolonged to allow for the definition of the optimal treatment period and determination whether 8% pirfenidone gel has met the objectives for patients with localized scleroderma (that is, by improving the lesions, delaying their progress and preventing permanent disability and cosmetic damage) [22,24]. We consider that pirfenidone can be a promising therapeutic agent for localized scleroderma.

## Conclusions

This is the first study on the therapeutic use of pirfenidone gel in localized scleroderma, showing response clinically with the mLoSSI, durometer and histological evidence. Considering its effectiveness, good safety profile and the advantage of topical application, pirfenidone 8% gel is a treatment option in localized scleroderma.

### Consent

Written informed consent was obtained from the patients for publication of their individual details and any accompanying images. The consent forms are held by the authors’ institution and are available for review by the Editor-in-Chief of this journal.

## Abbreviations

CTGF: Connective tissue growth factor
DU: Durometer unit
IL-10: Interleukin 10
mLoSSI: Modified Localized Scleroderma Skin Severity Index
PDGF: Platelet-derived growth factor
TGF-β: Transforming growth factor β
TNF-α: Tumor necrosis factor α
VAS: Visual analogue scale
